# Electrochemical analysis of the corrosion inhibition effect of trypsin complex on the pitting corrosion of 420 martensitic stainless steel in 2M H_2_SO_4_ solution

**DOI:** 10.1371/journal.pone.0195870

**Published:** 2018-04-19

**Authors:** Roland Tolulope Loto

**Affiliations:** Department of Mechanical Engineering, Covenant University, Ota, Ogun State, Nigeria; Duke University Marine Laboratory, UNITED STATES

## Abstract

Inhibition effect of trypsin complex (TC) on the pitting corrosion of martensitic stainless steel (type 420) in 1M H_2_SO_4_ solution was studied with potentiodynamic polarization, open circuit potential measurement and optical microscopy. TC reduced the corrosion rate of the steel with maximum inhibition efficiency of 80.75%. Corrosion potential shifted anodically due to the electrochemical action of TC. The pitting potential increased from 1.088V_Ag/AgCl (3M)_ at 0% TC to 1.365V_Ag/AgCl(3M)_ at 4% TC. TC shifts the open circuit corrosion potential from -0.270s at 0% TC concentration to -0.255V at 5% TC. The compound completely adsorbed onto the steel according to Langmuir, Frumkin and Temkin isotherms. ATF-FTIR spectroscopy confirmed the inhibition mode to be through surface coverage. Thermodynamic calculations showed physisorption molecular interaction. Corrosion pits are present on the uninhibited 420 morphology in comparison to TC inhibited surface which slightly deteriorated.

## Introduction

Stainless steels have extensive applications in chemical processing, petrochemical plants, waste water treatment plants etc. due to resistance to corrosion. The corrosion resistance of stainless steel in aqueous environments is due to an adherent, invisible and passive oxide film on the steel’s surface consisting of a chromium (III) oxide inner barrier and iron-rich outer deposited hydroxide or salt layer [1–4]. However they do suffer from the effect of localized corrosion attack. Pitting corrosion is considered the most destructive form of corrosion due to the difficulty in predicting its occurrence especially when the concentration of corrosive anions in aqueous environment increases. The location of corrosion pits on stainless steels is often unpredictable as pits tend to randomly disperse on the surface with preference for sites with non-metallic inclusions. One of the most important methods to prevent pitting of metallic alloys is the development of new corrosion resistant alloys with improved or reinforced metallurgical structures to withstand the effect of corrosive anions, but the major disadvantage is their high cost compared with conventional stainless steels and, as a result use of chemical compounds for corrosion inhibition is an effective and cheaper alternative. Some organic compounds have shown excellent inhibition performance adsorbing onto the steel’s surface through film formation [5–9]. Studies on the effect of sulphate anions on the passivation characteristics and pitting corrosion resistance of stainless steels are rare [10, 11]. This research aims to study the inhibiting effect of trypsin complex on 420 martensitic stainless steel in dilute H_2_SO_4_ media.

## Experimental methods

420 stainless steel (420SS) obtained commercially at Steel Works, Lagos, Nigeria has nominal (wt. %) composition of 13% Cr, 1% Si, 0.8% Mn, 0.04% P, 0.03% S, 0.15% C and 84.98% Fe. The stainless steel has a cylindrical shape with an exposed surface area of 0.79cm^2^. 420SS specimens were machined and grinded with silicon carbide papers (80, 320, 600, 800 and 1000) before washing with distilled water and acetone for potentiodynamic polarization test according to ASTM G1–03 [12]. Trypsin complex (TC) obtained from Bell, Sons & Co. Ltd, UK is a transparent oily liquid with a molar mass of 933.45g/mol and molecular formula of C_57_H_104_O_9_. TC was prepared in volumetric concentrations of 1%, 2%, 3%, 4% and 5% per 200mL of 1M H_2_SO_4_ acid prepared from analar grade (98%) with distilled water. Potentiodynamic polarization measurements were carried out at 30 °C using a three electrode system in an aerated glass cell containing 200mL of the prepared acid solutions at specific concentrations of TC and cylindrical 420SS electrodes with a Digi-Ivy 2311 potentiostat. Polarization plots were obtained at a scan rate of 0.0015V/s at potentials of -0.6V and +2.5V. Micro-analytical images of corroded and inhibited 420SS surface morphology were analysed after the polarization test with Omax trinocular metallurgical microscope.

## Result and discussion

### Potentiodynamic polarization studies

**Fig 1:**
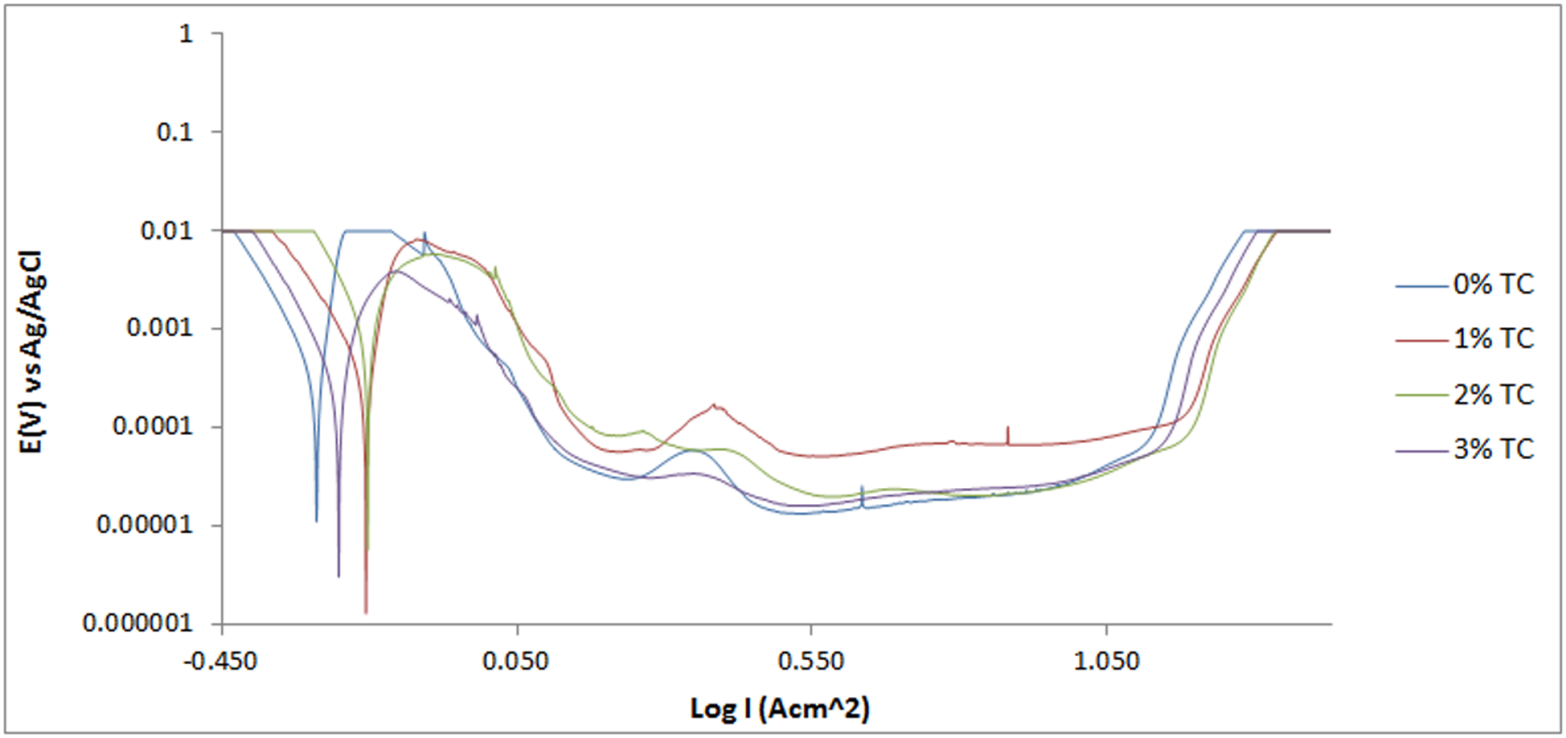
Potentiodynamic polarization curves of 420SS in 1M H_2_SO_4_/1%—3% TC solutions.

**Fig 2:**
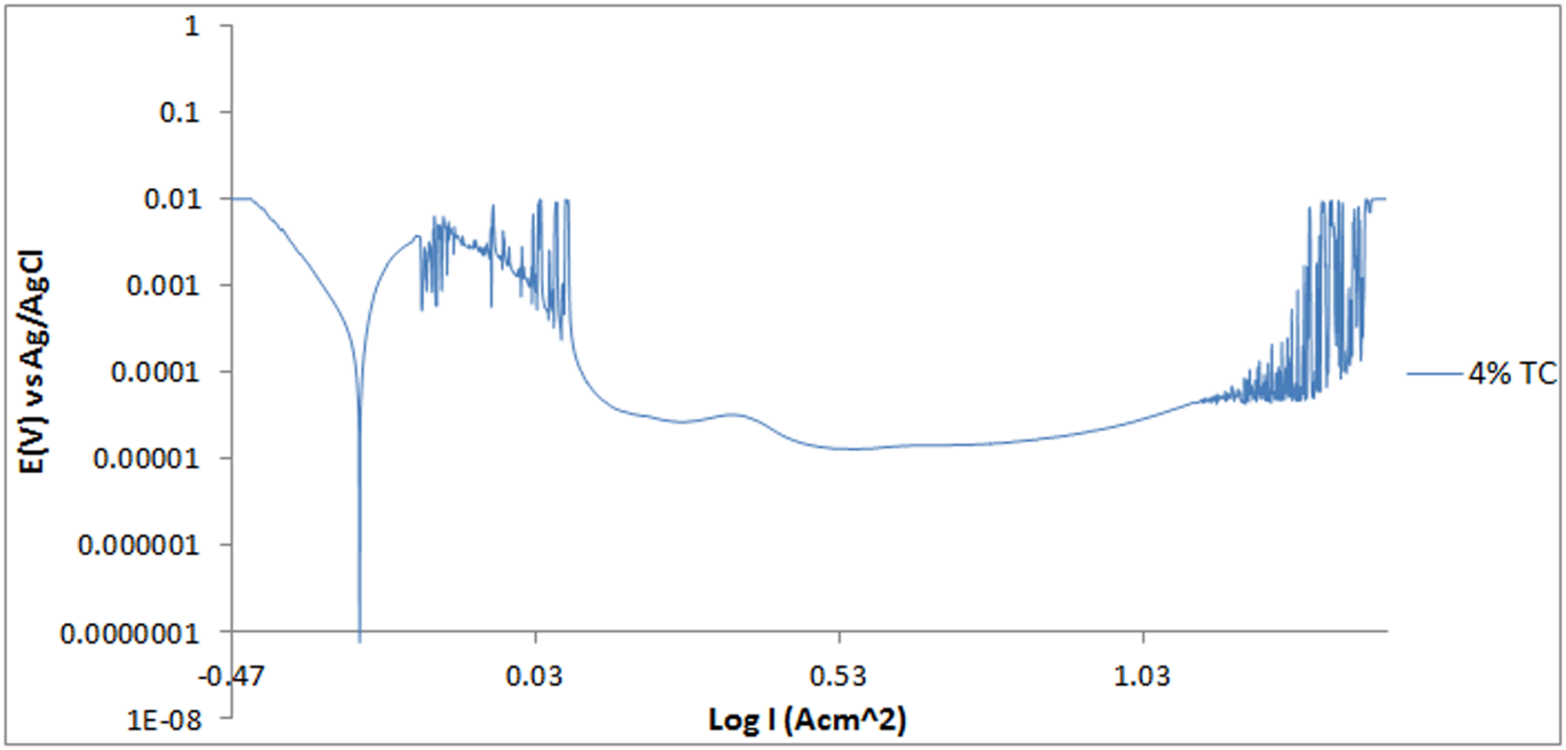
Potentiodynamic polarization curves of 420SS in 1M H_2_SO_4_/4% TC solutions.

**Fig 3:**
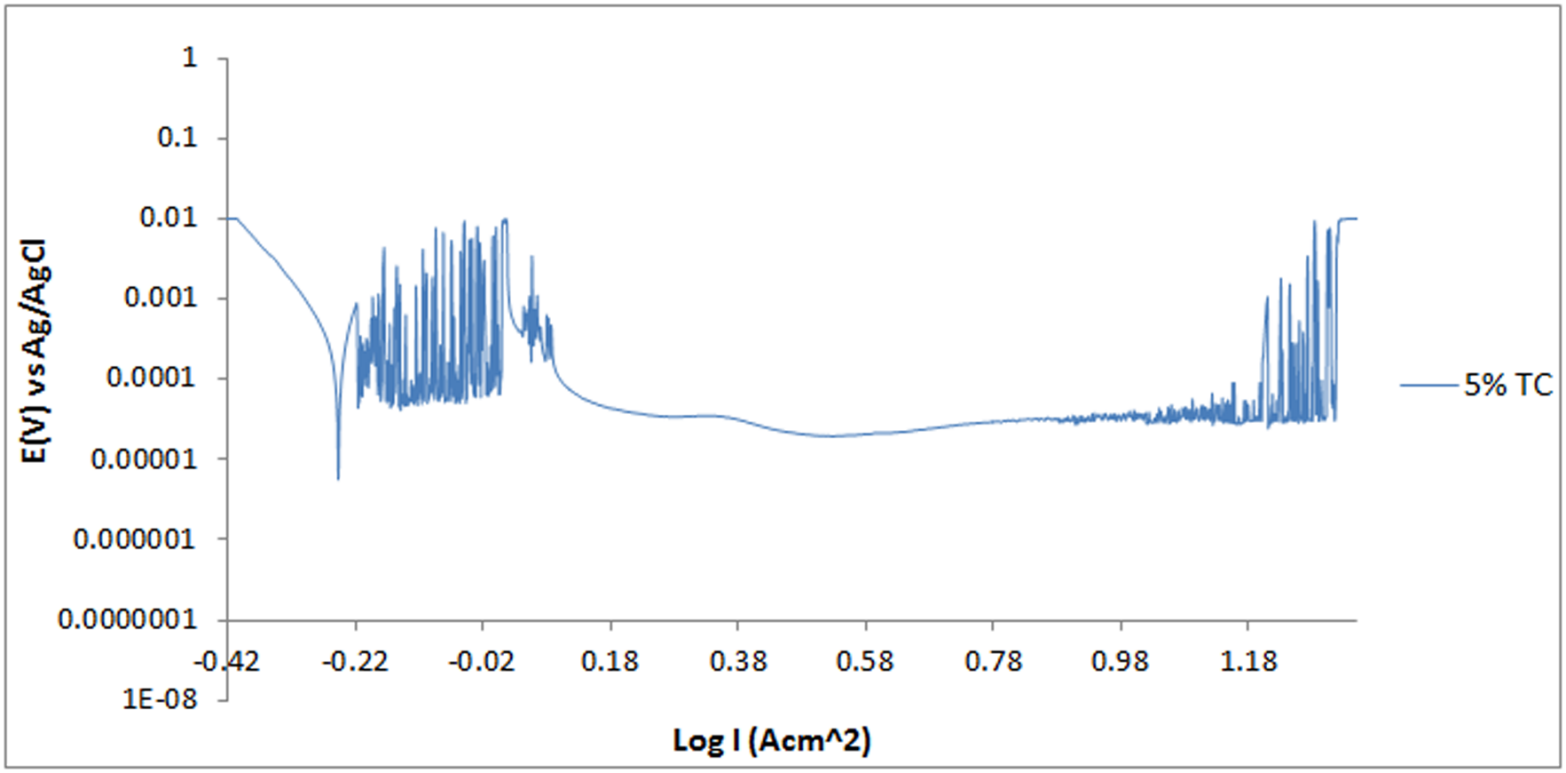
Potentiodynamic polarization curves of 420SS in 1M H_2_SO_4_/5% TC solutions.

The potentiodynamic polarization curves for 420SS electrodes in 3 M H_2_SO_4_ solution are shown in Figs 1–3. Table 1 shows the results for *C*_R_, *E*_cr_, *J*_cr_, *ɲ* and Tafel slope values. 420SS at 0% TC exhibited severe anodic dissolution and surface deterioration with corrosion rate value of 30.79mm/y due to the diffusion polarization of cathodic and anodic reactions resulting from the electrochemical action of SO_4_^2-^ anions in the acid solution. The SO_4_^2-^ anions weakened the passive film consisting of Cr_2_O_3_, at sites with impurities due to the effect of applied potential. This causes the release of ferrous ions, produced at the metal/passive oxide interface and migrates through the passive film to the oxide-solution interface. On the polarization plot for 420SS at 0% TC [Fig 1], increase in applied over-potential caused a corresponding increase in corrosion rate along the anodic-cathodic branch of the plot. The reaction phenomenon is associated with active behavior of the metal alloy. Beyond the anodic portion of the plot the corrosion rate drops by significant magnitudes due to the formation of a passive film on 420SS which extends over a wide potential till the applied potential eventually cause breakdown of the passive film resulting in a transpassive state and corrosion increase. Corrosion rate values for 420SS at 1%–5% TC concentration significantly contrast the value obtained at 0% TC due to the corrosion inhibiting action of TC organic molecules. Increase in TC concentration caused a proportionate decrease in corrosion rate till 6.72 mm/y at 5% TC. There was also a subsequent increase in the passive region of the polarization plot as TC molecules protect 420SS surface from the corrosive anions. Addition of TC shifts the corrosion potential of 420SS at 0% TC anodically to varying potentials values with respect to TC concentration, signifying anodic inhibition due to surface coverage. The limited change in cathodic Tafel slopes values with TC addition to the acid solution confirms that TC dominantly hindered the oxidation reaction responsible for metal dissolution. However the higher cathodic Tafel slope values confirm significant O_2_ reduction reactions. The cathodic polarization of the metal alloy in the electrolyte confirms cathodic protection whereby the cathodic potential is toward the negative direction of the potential shift. The anodic slope changed in value between TC inhibited and uninhibited 420SS electrodes. The anodic Tafel value at 0% TC is the product of oxide formation on the 420SS surface due to breakdown of the passive film, resulting from the slow electron transfer step. The maximum change in corrosion potential is 87mV in the anodic direction thus TC is an anodic type inhibiting compound [13].

**Table 1:** Potentiodynamic polarization results for 420SS in 1M H_2_SO_4_/0%—5% TC solution.

Sample	TC Conc. (%)	TC Conc. (M)	Corrosion Rate (mm/y)	TC Inhibition Efficiency	Corrosion Current (A)	Corrosion Current Density (A/cm^2^)	Corrosion Potential (V_Ag/AgCl_)	Polarization Resistance, *R*_p_ (Ω)	Cathodic Tafel Slope, *B*_c_ (V/dec)	Anodic Tafel Slope, *B*_a_ (V/dec)
A	0	0	30.79	0	2.24E-03	2.83E-03	-0.290	32.34	-10.660	0.015
B	1	1.07E-02	12.31	60.02	8.94E-04	1.13E-03	-0.206	35.43	-8.627	2.947
C	2	2.14E-02	9.98	71.42	7.25E-04	9.18E-04	-0.203	13.99	-10.630	2.632
D	3	3.21E-02	7.88	77.43	5.73E-04	7.25E-04	-0.257	44.87	-11.400	5.601
E	4	4.29E-02	7.14	79.56	5.19E-04	6.56E-04	-0.265	49.54	-10.310	5.213
F	5	5.36E-02	6.72	80.75	4.88E-04	6.18E-04	-0.252	32.59	-9.796	3.025

### Pitting corrosion evaluation

Under anodic polarization the 420SS samples acquired a passive state, with breakdown at the pitting potential [Figs 1–3]. The polarization curve of 420SS at 0% TC concentration passivated at 0.102V_Ag/AgCl_ following metastable pitting activity to pit at 1.088V_Ag/AgCl_. The presence of SO_4_^2-^ anions significantly aggravates the conditions for formation and growth of the pits on the stainless steel through an autocatalytic process resulting in relatively low pitting corrosion resistance. The pitting potential value referred to earlier is the result of breakage or dissolution of the Cr(III) oxide layer of the stainless steel causing pitting corrosion [14]. Addition of TC compound (1%–5%TC) increased the potential at which pitting occurs (Table 2), while simultaneously reducing the metastable pitting current due to the inhibiting action of TC molecules. TC invariably reinforced the corrosion resistance exhibited by the Cr(III) oxide hydroxides present in the passivating layers of the 420SS surface, hence increasing the pitting corrosion resistance of the steel. The concentration of SO_4_^2-^ anions reaching the steel’s surface is considerably reduced. The passivation range of 1–5% TC inhibited the 420SS surface over a wider potential range in comparison to the potential range of 420SS at 0% TC as earlier mentioned. At 4%–5% TC current transients are visible on the polarization curves due to the active-passive behavior of the protective film on the steel surface during metastable pitting activity and towards the end of the potential range for passivity. The surface film of the steel towards the end of the passivated regions is less stable, and further growth of the film was hindered, and eventually film breakdown under relatively high applied potential occurs.

**Table 2:** Potentiostatic data for 420SS in 1M H_2_SO_4_/0%—5% TC solution.

Sample	TC Conc. (M)	Pitting Potential, *E*_pitt_ (V)	Passivation Potential, *E*_p_ (V)	Passivation Range (V)	Metastable Pitting Current (A)
A	0	1.088	0.102	0.986	9.94E-03
B	1	1.139	0.143	0.996	5.06E-03
C	2	1.149	0.150	0.999	4.75E-03
D	3	1.089	0.105	0.984	2.72E-03
E	4	1.365	0.103	1.262	2.58E-03
F	5	1.293	0.091	1.202	6.79E-04

### Open circuit potential measurement

**Fig 4:**
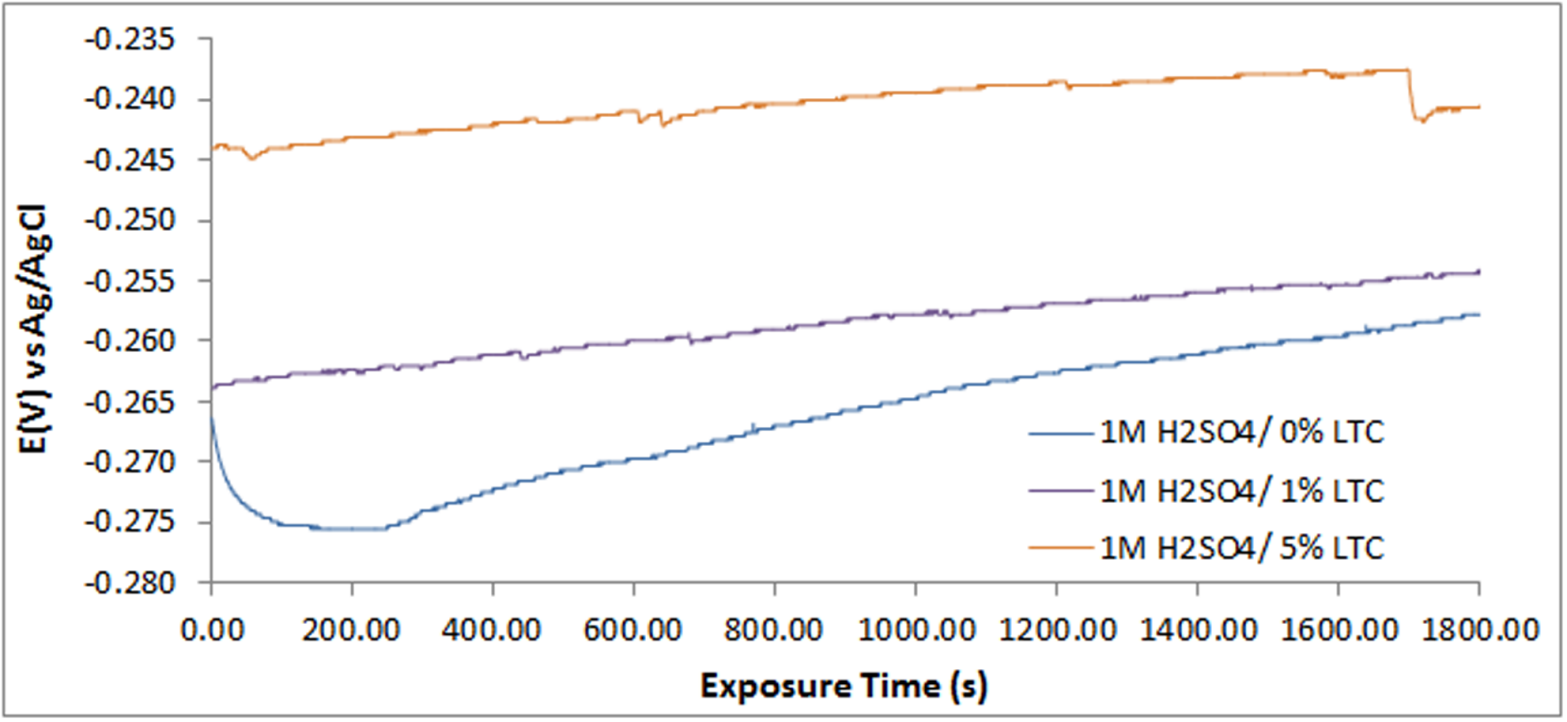
Variation of OCP values versus exposure time.

Open circuit potential measurement (OCP) plots for 420SS samples in 1M H_2_SO_4_/0%, 1% and 5%TC solutions are shown in Fig 4. Plots for 420SS in 1M H_2_SO_4_/0% TC decreased from -0.270V_Ag/AgCl_ at 0s to -0.275V_Ag/AgCl_ at 82.05s due to corrosion resulting from the electrochemical action of SO_4_^2-^ anions on 420SS surface, as the passive protective film forms on the steel. Between 82.05s and 232.15s the OCP values remained generally constant before increasing consistently to 1800s due to increased passivation of the steel. The passivation behavior of 420SS in 1M H_2_SO_4_/1% TC contrast the plot produced in 1M H_2_SO_4_/0%. The presence of TC at 1% concentration shifts the OCP value of 420SS to -0.265V_Ag/AgCl_ at 0s due to the passivation effect of TC, and the OCP value progressively increased till 1800s. Increasing the concentration of TC to 5% TC significantly increased the passivation of 420SS to -0.244V_Ag/AgCl_ at 0s. This observation confirms the concentration dependent corrosion inhibition performance and passivation characteristics of TC compound.

### Optical microscopy analysis

**Fig 5:**
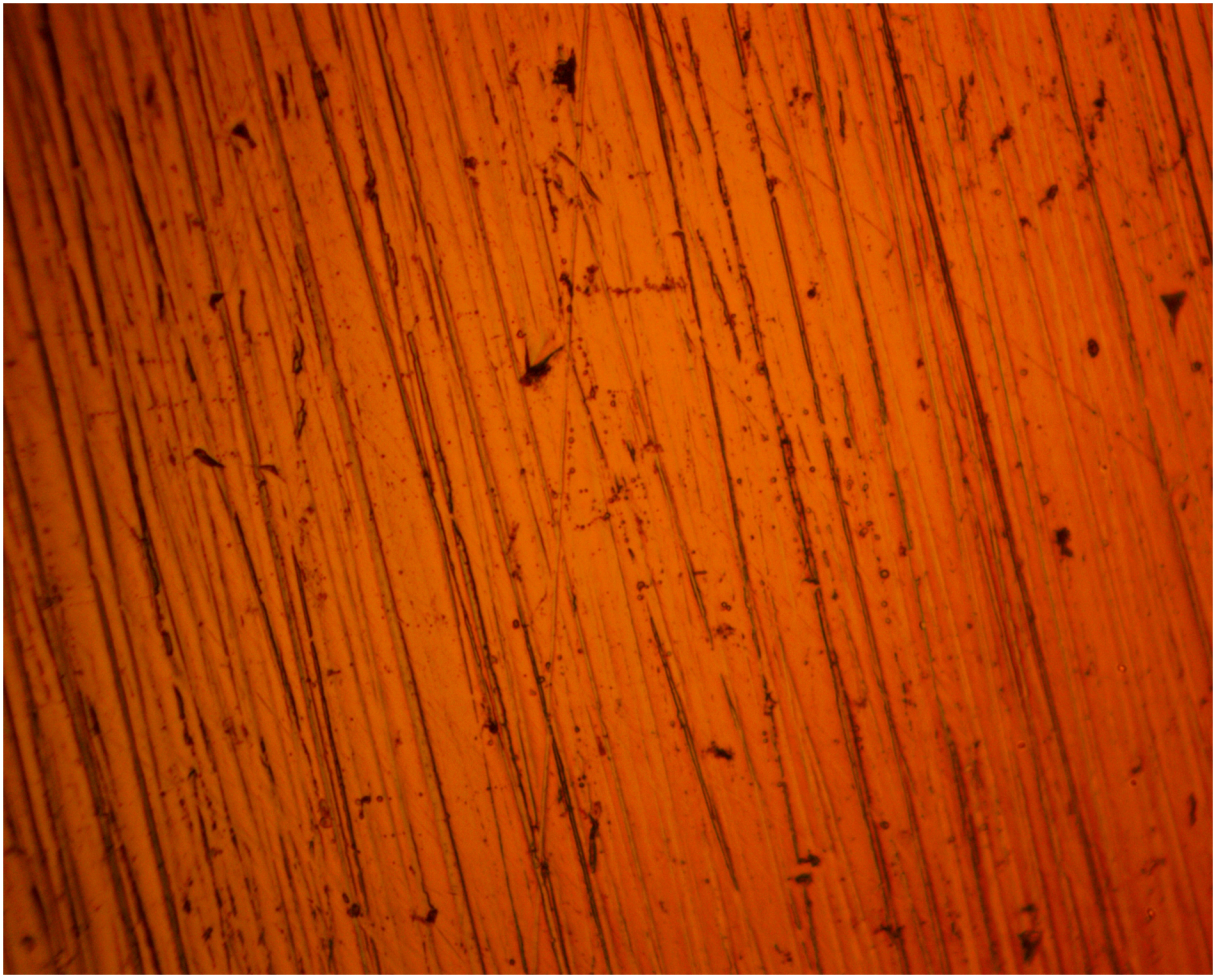
Micro-analytical image of 420SS (mag. x40) before corrosion test.

**Fig 6:**
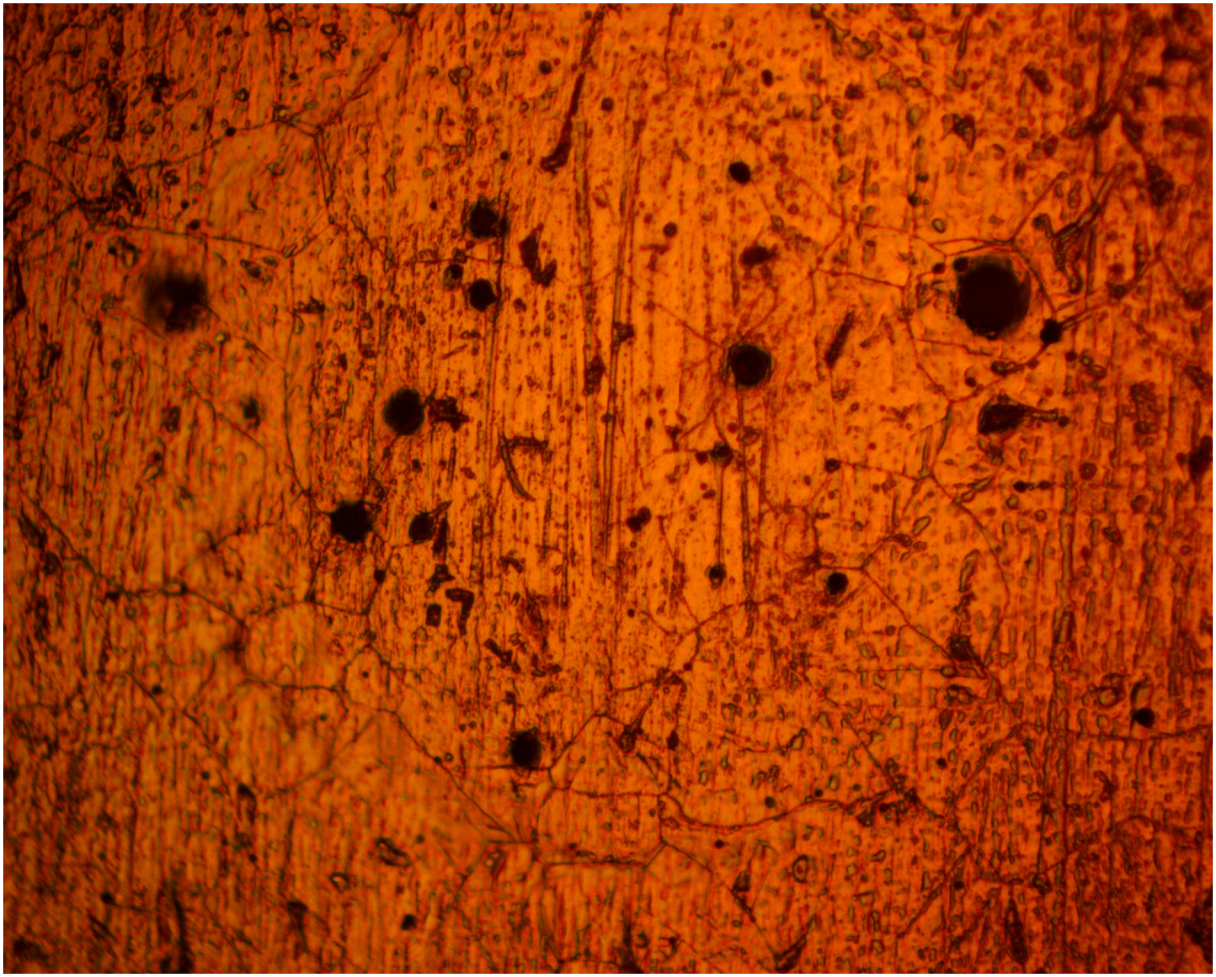
Micro-analytical image of 420SS (mag. x40) after corrosion without TC compound in 1M H_2_SO_4_.

**Fig 7:**
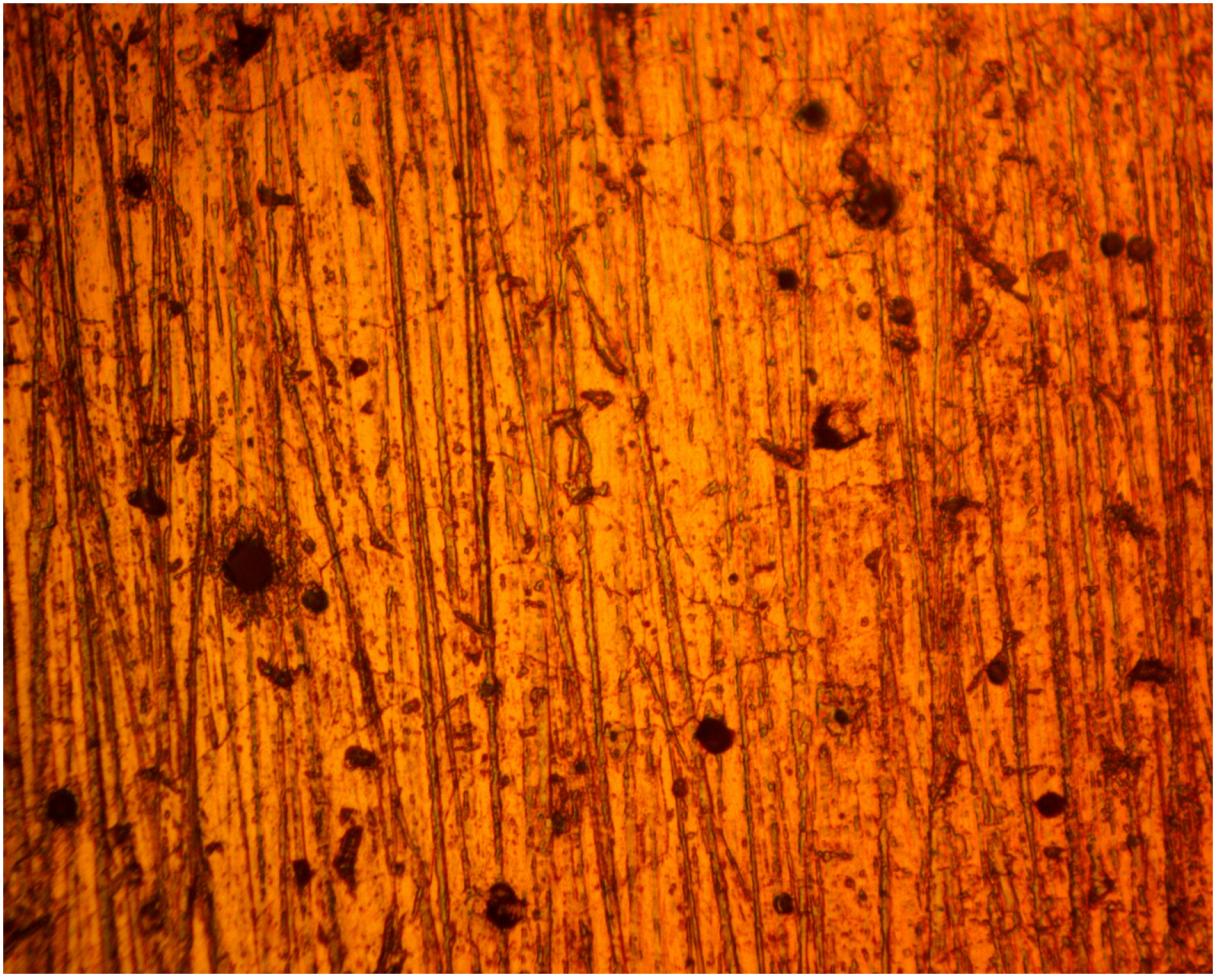
Micro-analytical image of 420SS (mag. x40) after corrosion in 1% TC/1M H_2_SO_4_.

**Fig 8:**
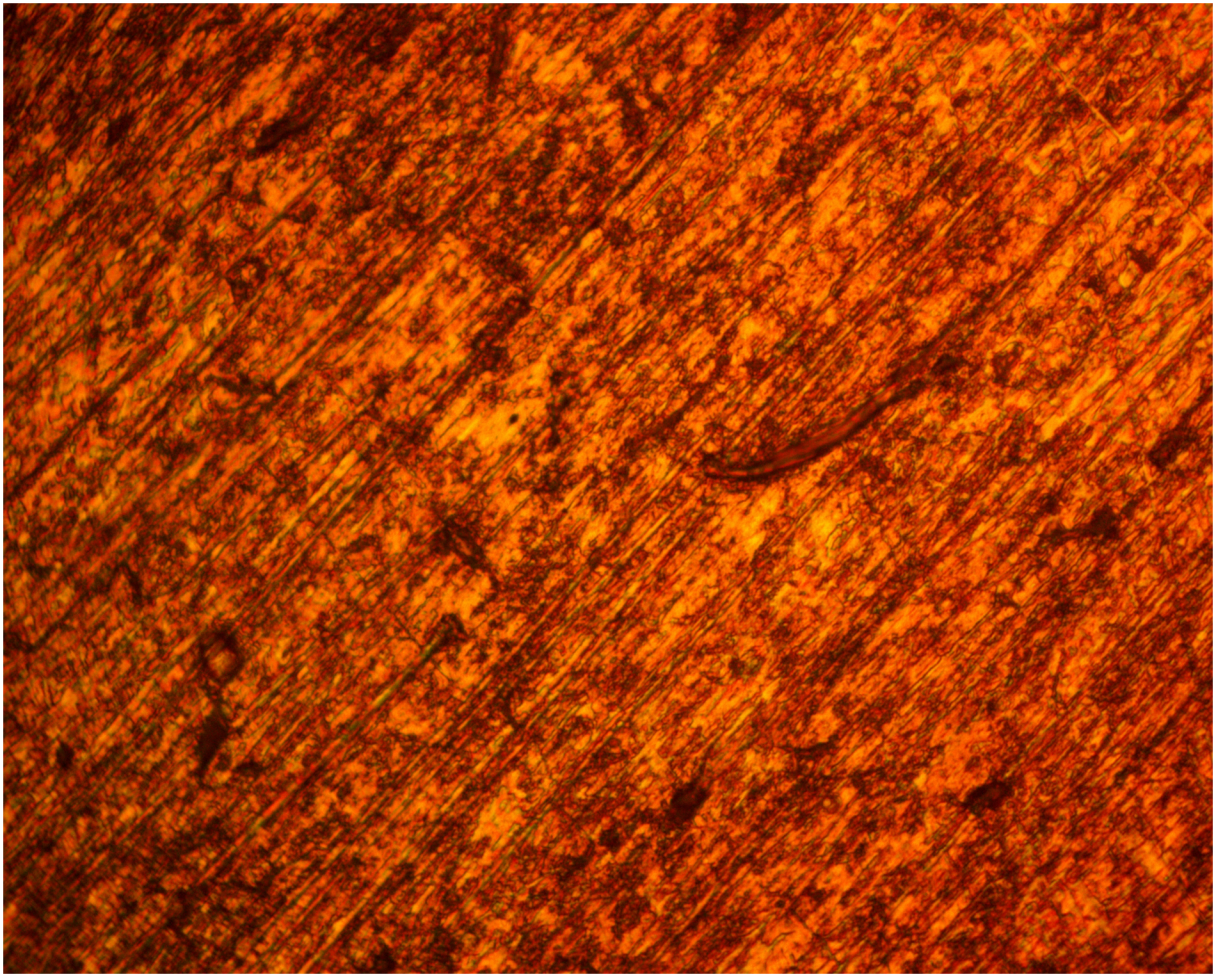
Micro-analytical image of 420SS (mag. x40) after corrosion in 5% TC/1M H_2_SO_4_.

Figs 5 and 6 shows the morphology of 420SS before corrosion and after corrosion without TC addition, while Figs 7 and 8 shows the morphology of 420SS after corrosion in 1% and 5% TC/1M H_2_SO_4_ solution. The morphology of 420SS in Fig 6 shows a badly corroded and worn-out surface with numerous micro/macro pores and corrosion pits due to the electrochemical action of SO_4_^2-^ anions on the steel’s surface. The corrosion resistance of 420SS is known to arise from the high corrosion resistance exhibited by the Cr(III) oxide-hydroxides layer [15], hence the pitted sites in Fig 6 represent breakage of the passive film. Observation of Fig 7 shows an improvement in the general features of 420SS compared to Fig 5, Corrosion pits are also visible but appears to be smaller and shallower. Fig 8 shows a well inhibited surface void of corrosion pits due to the electrochemical action of TC molecules at 5% TC concentration.

### Adsorption isotherm studies

**Fig 9:**
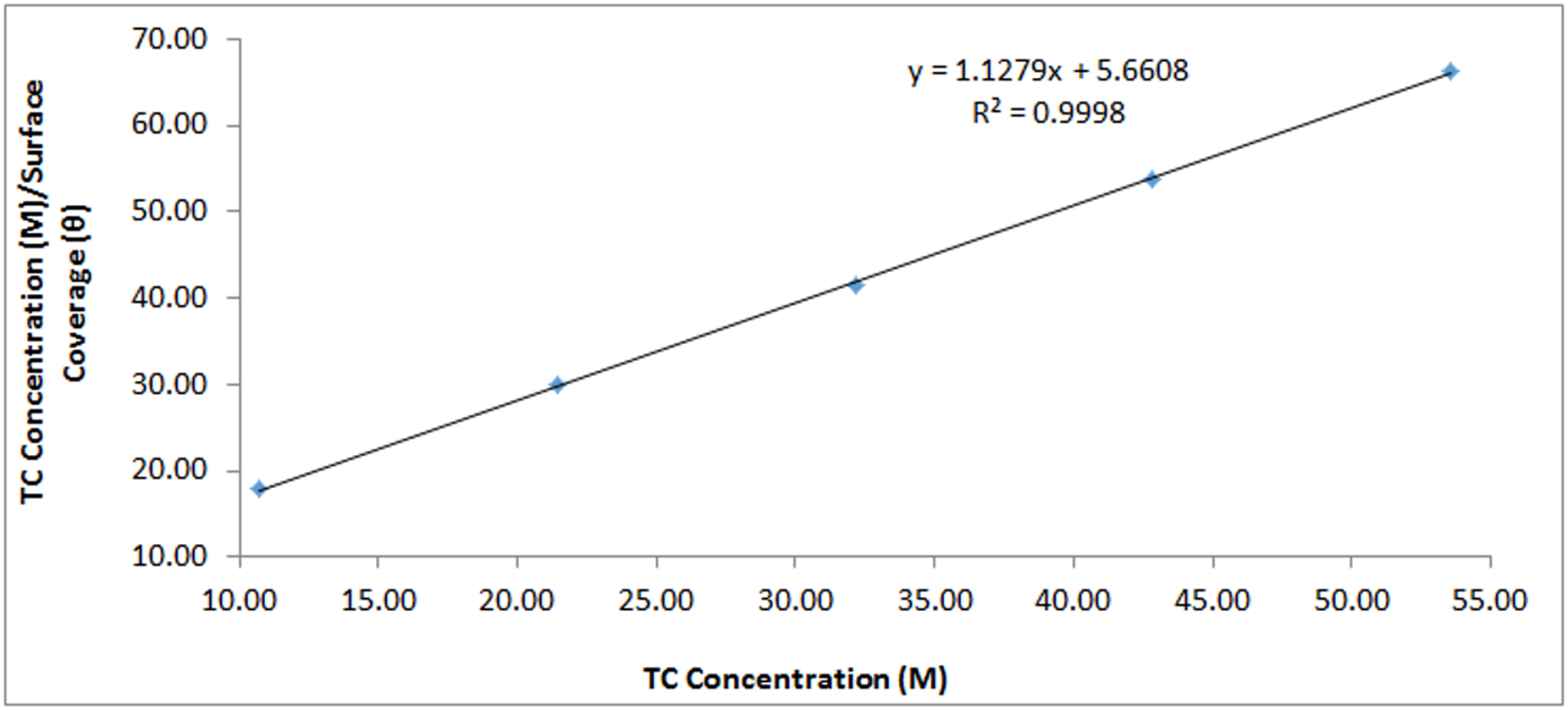
Langmuir isotherm plot of $\frac{C_{LTC}}{\theta }$ versus LTC concentration in 1M H_2_SO_4_.

Langmuir, Frumkin and Temkin isotherms showed the best fitting for TC adsorption on 420SS from correlation coefficient values obtained [16]. The Langmuir isotherm states that the presence of definite amount of vacant adsorption sites on the metallic surface are of equal dimension and shape with a specific amount of inhibitor molecule. As a result specific amount of energy is released and there is no lateral interaction between the adsorbed inhibitor molecules [17]. Fig 9 shows the plots of $\frac{C_{TC}}{\theta }$ vs *C*_TC_ with a correlation coefficient of 0.9998 according to the Langmuir equation.

$$\theta =[\frac{K_{TC}C_{TC}}{1+K_{TC}C_{TC}}]$$ (1)

*θ* is the amount of TC adsorbed per unit gram on 420SS surface at equilibrium. *C*_TC_ is TC inhibitor concentration and *K*_TC_ is the equilibrium constant of adsorption. Frumkin isotherm states metallic surfaces are heterogeneous and the lateral interaction effect among adsorbed TC molecules is not negligible according to Eq 2:

$$\frac{\theta }{1-\theta }=K_{TC}{Ce}^{2\alpha \theta }$$ (2)

**Fig 10:**
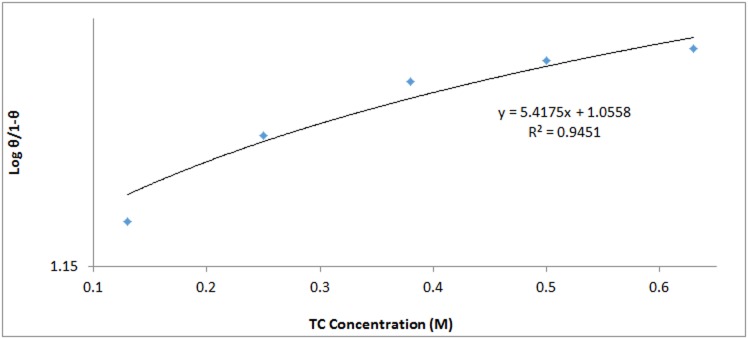
Frumkin isotherm plot of $log[\frac{\theta }{(1-\theta )C}]$ versus *θ* in 1M H_2_SO_4_.

α is the interaction parameter which describes the molecular interaction in adsorbed layer, and calculated from the slope of the Frumkin isotherm plot. *K* is the adsorption-desorption constant. Plots of $log[\frac{\theta }{(1-\theta )C}]$ versus *θ* in Fig 10 showed a correlation coefficient of 0.9451 in H_2_SO_4_ solution. The Temkin isotherm assumes the heat of adsorption decreases linearly with increase in surface coverage according to the equation

qe=BIn(A+Ce) (3)

Where

$$B=\frac{RT}{b}$$ (4)

**Fig 11:**
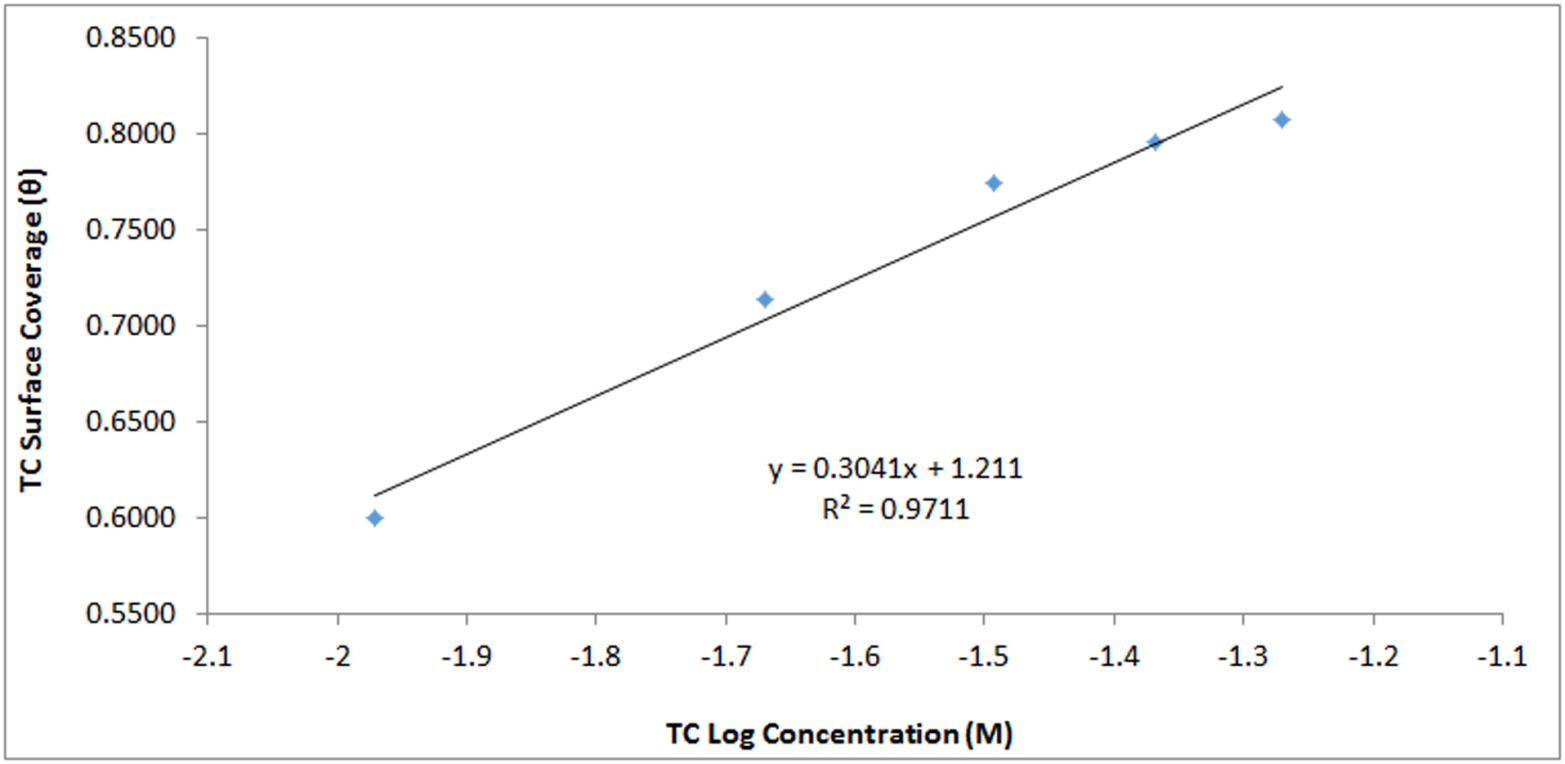
Temkin isotherm plot of TC surface coverage versus TC Log concentration in 1M H_2_SO_4_.

*A* is Temkin isotherm constant (L/g), b is the Temkin constant related to heat of adsorption, *T* is the temperature (K), *R* is the gas constant (8.314, J/mol.K) and Ce is the concentration of adsorbate. *B* is the Temkin constant related to heat of sorption (J/mol). The Temkin isotherm plot for TC adsorption in H_2_SO_4_ Fig 11 has a correlation coefficient of 0.9711.

### Thermodynamics of the corrosion inhibition mechanism

Calculated results of Gibbs free energy of adsorption in H_2_SO_4_ solution is shown in Table 3, from Eq 5.

$$\Delta G_{ads}=-2.303\;RTlog\;[55.5\;K_{LTC}]$$ (5)

where 55.5 is the molar concentration of water in the acid solution, *R* is the universal gas constant, *T* is the absolute temperature and *K*_TC_ is the equilibrium constant of TC adsorption on 420SS. The negative values of Δ*G*^*o*^_ads_ results show the spontaneity and stability of the adsorption mechanism. The highest Δ*G*^*o*^_ads_ value is -22.20KJmol^-1^ at the lowest TC concentration; while the lowest Δ*G*^*o*^_ads_ value is -20.76KJmol^-1^ at the highest TC concentration on 420SS surface due to the effect of lateral repulsion among LTC molecules at higher TC concentration. The Δ*G*^*o*^_ads_ values shows physisorption adsorption mechanisms on 420SS surface, confirming earlier assumption that the inhibition mode is through surface coverage [18, 19].

**Table 3:** Results for Gibbs free energy (Δ*G*^*o*^_ads_), surface coverage (θ) and equilibrium constant of adsorption (*K*_ads_) for TC adsorption on 420SS in 1M H_2_SO_4_ solution.

Specimen	TC Concentration (*M*)	Surface Coverage (*θ*)	Equilibrium Constant of adsorption (*K*)	Gibbs Free Energy, ΔG (Kjmol^-1^)
A	0	0	0	0
B	1.07E-02	0.600	140.1	-22.20
C	2.14E-02	0.714	116.6	-21.75
D	3.21E-02	0.774	106.7	-21.53
E	4.29E-02	0.796	90.8	-21.13
F	5.36E-02	0.808	78.3	-20.76

### ATF-FTIR spectroscopy

**Fig 12:**
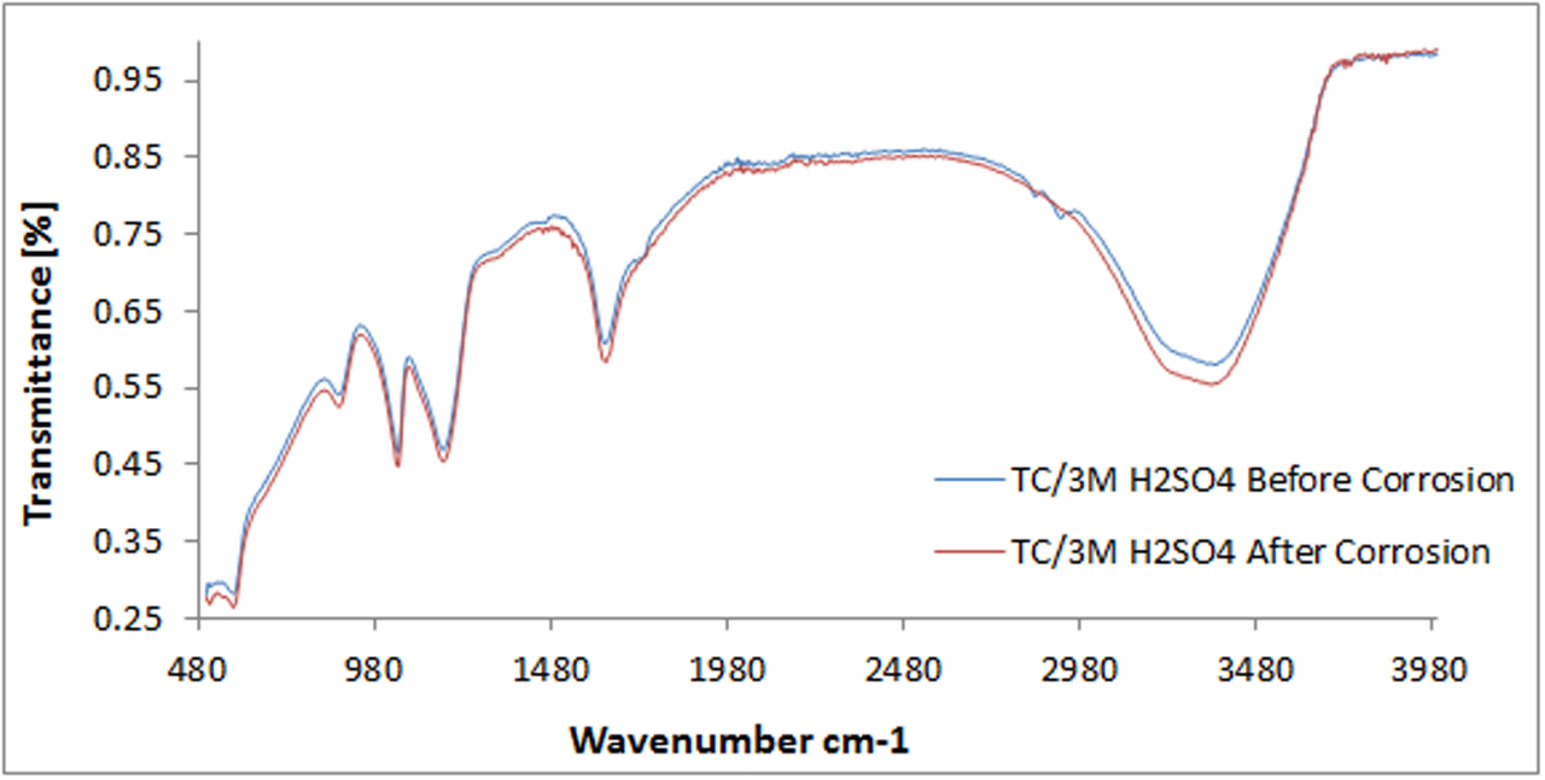
ATF-FTIR spectra of TC compound in 3M H_2_SO_4_ solution before and after 420SS corrosion.

The spectra diagram for TC adsorption and corrosion inhibition of 420SS in 3M H_2_SO_4_ is shown in Fig 12. The spectra plots of TC/3M H_2_SO_4_ before and after corrosion showed similar configuration. There are no significant changes in wavelength values except, between 3181.86cm^-1^ and 3469.45cm^-1^ where there is a noticeable decrease in the spectra plot for TC/3M H_2_SO_4_ after corrosion due to adsorption of the functional groups consisting of alcohols, phenols, primary and secondary amines, amides, carboxylic acids and alkynes terminals. Limited adsorption seems to have occurred at the earlier mentioned wavelengths but its overall effect is negligible as the similarity in the total peak configuration shows that surface coverage through physisorption adsorption mechanism is majorly responsible for corrosion inhibition. Breakage of the protective film covering is responsible for pitting of the steel surface.

## Conclusion

The inhibition effect of trypsin complex on the pitting corrosion resistance of 420 martensitic stainless steel was investigated in dilute sulphuric acid. Comparison of polarization plots and results obtained showed the trypsin complex significantly increase the potential necessary for pit initiation and the passivation range of the steel, thus improving the steel’s resistance to pitting corrosion. The compound formed an impenetrable protective film on the steel surface through physisorption mechanism according to Langmuir, Frumkin and Temkin adsorption isotherms. Corrosion pits on the uninhibited steel surface resulting from the electrochemical action of sulphate anions were absent on the inhibited steel morphology due to the effective inhibiting action of trypsin complex.
